# How vital are the vital signs? a multi-center observational study from emergency departments of Pakistan

**DOI:** 10.1186/1471-227X-15-S2-S10

**Published:** 2015-12-11

**Authors:** Amber Mehmood, Siran He, Waleed Zafar, Noor Baig, Fareed Ahmed Sumalani, Juanid Abdul Razzak

**Affiliations:** 1 Johns Hopkins International Injury Research Unit, Department of International Health, Johns Hopkins Bloomberg School of Public Health, Baltimore, Maryland, USA; 2 Department of Emergency Medicine, Aga Khan University, Karachi, Pakistan; 3 Department of Emergency Medicine, Sandamen provincial Hospital(Civil Hospital), Quetta, Pakistan; 4 Department of Emergency Medicine, John Hopkins School of Medicine, Baltimore, Maryland, USA; 5 The author was affiliated with the Department of Emergency Medicine, Aga Khan University, Karachi, Pakistan at the time when study was conducted

**Keywords:** Emergency Department, Triage, Guidelines, Surveillance, Pakistan

## Abstract

**Background:**

Vital signs play a critical role in prioritizing patients in emergency departments (EDs), and are the foundation of most triage methods and disposition decisions. This study was conducted to determine the frequency of vital signs documentation anytime during emergency department treatment and to explore if abnormal vital signs were associated with the likelihood of admission for a set of common presenting complaints.

**Methods:**

Data were collected over a four-month period from the EDs of seven urban tertiary care hospitals in Pakistan. The variables included age, sex, hospital type (government run vs. private), presenting complaint, ED vital signs, and final disposition. Patients who were >12 years of age were included in the analysis. The data were analyzed to describe the proportion of patients with documented vitals signs, which was then crossed-tabulated with top the ten presenting complaints to identify high-acuity patients and correlation with their admission status.

**Results:**

A total of 274,436 patients were captured in the Pakistan National Emergency Department Surveillance (Pak-NEDS), out of which 259,288 patients were included in our study. Vital signs information was available for 90,569 (34.9%) patients and the most commonly recorded vitals sign was pulse (25.7%). Important information such as level of consciousness was missing in the majority of patients with head injuries. Based on available information, only 13.3% with chest pain, 12.8% with fever and 12.8% patients with diarrhea could be classified as high-acuity. In addition, hospital admission rates were two- to four-times higher among patients with abnormal vital signs, compared with those with normal vital signs.

**Conclusion:**

Most patients seen in the EDs in Pakistan did not have any documented vital signs during their visit. Where available, the presence of abnormal vital signs were associated with higher chances of admission to the hospital for the most common presenting symptoms.

## Background

Emergency departments (EDs) prioritize care such that the most critically ill and injured patients receive care first [1]. In many instances, EDs also provide unscheduled care for a wide variety of acute conditions that could be dealt with in a primary care setting [2-4]. This makes prioritization of care an integral part of the ED gatekeeping, and in many developed countries, this is a function of triage to expedite patient care, streamline resources, and in some cases facilitate timely disposition [5,6]. Combinations of the presenting complaint, vital signs, and selected patient characteristics such as age and pre-existing medical conditions can be combined to generate algorithms defining the urgency of the clinical condition and potential interventions by the health care providers [7]. Initial vital signs can help the triage decisions, allocate resources, and even patients' ED disposition[8]. Literature also supports that a combination of presenting complaint with the initial vital signs could be highly predictive of both an intensive care unit (ICU) stay and in-hospital mortality [9].

Some studies from Pakistan highlight the problem of ED overcrowding and patients leaving the ED without being seen, but there is limited information on the process of decision-making in the EDs of Pakistan [10,11]. The purpose of this study is to use the Pakistan National Emergency Department Study (Pak-NEDS) data to: 1) determine the number of ED patients for whom the vital signs were obtained 2) understand if vital signs can be utilized to identify high- acuity patients and 3) determine if there is a correlation between abnormal vital signs and ED disposition.

## Methods

### Study Design and Setting

Pak-NEDS was conducted from November 2010 to March 2011 to determine the burden of the patients presenting to large urban EDs and the pattern of diseases. Pak-NEDS was conducted at seven sites nationally. These sites included EDs of major tertiary-care hospitals across four provinces of Pakistan (Sindh, Punjab, Khyber Pakhtunkhwa, and Balochistan) and the federal capital, Islamabad. The participating institutes were the Aga Khan University, Karachi; Jinnah Post-Graduate Medical Center, Karachi; Benazir Bhutto Hospital, Rawalpindi; Mayo Hospital, Lahore; Lady Reading Hospital, Peshawar; Sandeman Provincial Hospital, Quetta; and Shifa International Hospital, Islamabad. Of the seven hospitals, five were government run hospitals and two were privately run and funded. The study was approved by the institutional review boards of each of the participating hospitals. More detailed description of Pak-NEDS methodology can be found in the supplement.

### Study Population and Outcome Measurements

All patients presenting to one of the participating EDs during the study period were registered in Pak-NEDS. Data collectors, who were present in EDs round the clock for this study, interviewed each patient (or their next of kin) and gathered clinical information through ED records to complete the data collection tool. The variables recorded included patient characteristics such as age, sex, mode of arrival, presenting complaint, recent history of trauma, first vital signs recorded in ED, Glasgow Coma Score (GCS), visual pain scale, providers' diagnosis, treatment, disposition, and diagnostic procedures done in EDs.

Several additional variables were defined for the purpose of analysis, including the following: Normal vital signs were defined a priori as blood pressure (120/80 mm/Hg to 90/60 mm/Hg); respiratory rate (12-18 breaths per minute); pulse (60-100 beats per minute); and temperature (36.5°C to 37.2°C, or 97.8 °F to 99.1 °F) [12]. For mental status assessment, a GCS score of <12 was considered clinically significant depressed mental function, or in case of trauma it indicates moderate to severe head injury; scores between13-15 were defined as mild head injury in the context of trauma [13-15]. To identify patients with hypovolemia, the value of the shock index (SI, which is the ratio of pulse rate to systolic blood pressure) was calculated in suitable clinical scenario. An SI within the range of 0.5-0.7 was considered normal; a higher ratio (SI> 0.7) was selected as a measure of hemodynamic instability secondary to volume loss or hemorrhage commonly observed in patients with severe diarrhea or vomiting, severe sepsis, and trauma [16].

### Statistical analysis

Data were entered and cleaned by a designated data entry team at Aga Khan University (AKU) using EpiInfo™ v.3.3.2 (Centers for Disease Control and Prevention, Atlanta, USA). The analyses were done using Stata^® ^v.12 (StataCorp LP, Texas, USA). For this study, all patients aged 12 years or below were excluded from the analysis to minimize variation in the range of normal vital signs. Over the study period of four months, data on 274,436 patients was collected through Pak-NEDS. This study sample consisted of 259,288 (94.5%) patients who were >12 years of age. All analyses were descriptive in this article.

First, general socioeconomic characteristics, namely sex, age group and nature of hospital (public or private) were tabulated for all patients over 12 years of age, and for patients with triage information recorded in this study. Wilson procedure for all proportions was used to generate 95% confidence intervals, and only significantly different intervals were presented. Second, the top ten presenting complaints were identified through comparison of their percentages in the sample, which was presented as a stacked bar graph with disposition information. Important vital signs were then crossed-tabulated with these ten presenting complaints to identify high-acuity patients. For instance, in patients with fever, vital signs such as temperature, pulse, systolic blood pressure, and GCS are considered important to be matched with the symptom, whereas for patients with shortness of breath, respiratory rate, pulse oximetry, and blood pressure measurements are considered vital to identify high-acuity patients. Finally, through cross-tabulation, this paper presents the comparison of the vital signs with patients' ED disposition for the top ten presenting symptoms.

## Results

**Figure 1:**
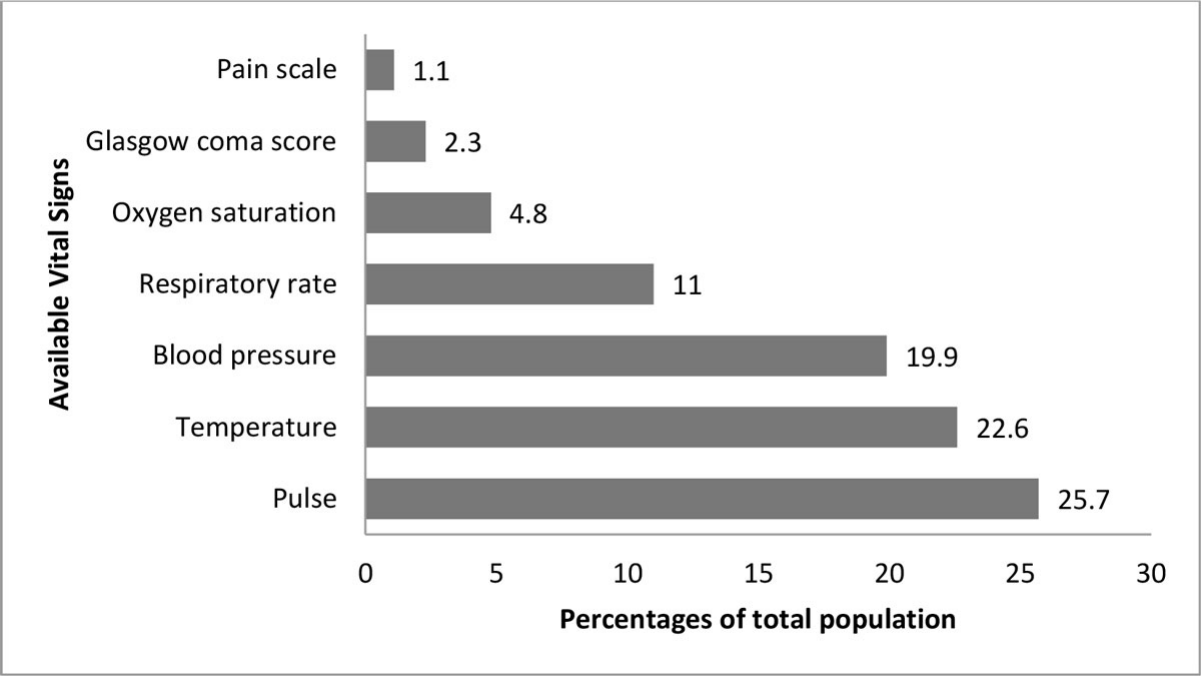
**Available vital signs for Pakistan National Emergency Department Surveillance patients (n = 259,288)**.

Among 259,288 patients who were >12 years of age, 153, 298 (59.1%) were males. The most common age group to be seen in the ED was 25-44 years (47.6%), followed by 15-24 years (23.1%). Table 1 summarizes the demographic characteristics of our sample. The majority (76.5%, 95%CI = [76.3%, 76.6%]) of the subjects in our study were seen in the public sector hospitals. Overall, 90,569 (34.9%) patients had documented vital signs. The most commonly recorded vital signs were pulse 66,695 (25.7%), temperature 61,143 (23.6%), blood pressure 51,633 (19.9%), and respiratory rate 28,599 (11.0%). Oxygen saturation measured via pulse oximetry (12,450, 4.8%) and Glasgow coma score (GCS) (5,994, 2.3%), were infrequently recorded in the ED. A pain scale was available in 2,473 (1.1%) patients with various clinical conditions (Figure 1).

**Table 1:** General characteristics of study participants (patients >12 years)

Characteristics		Sample for current analysis (n = 259,288)		Patients with triage done (n = 72,789)	
		
		N	% [95% CI]	N	% [95% CI]
*Sex*	Male	153,298	59.1	41,996	57.7
	Female	99,303	38.3	29,760	40.9
	Missing	6,687	2.6	1,033	1.4
*Age Group*	12-14 y	3,136	1.2	818	1.1
	15-24 y	59,797	23.1	14,883	20.5
	25-44 y	123,421	47.6	35,458	48.7
	45-64 y	47,363	18.3	14,333	19.7
	65+ y	10,684	4.1	3,503	4.8
	Missing	14,887	5.4	3,794	5.2
*Hospital*	Public	198,318	76.5 [76.3, 76.6]	42,275	58.1 [57.7, 58.4]
	Private	60,970	23.5 [23.4, 23.7]	30,514	41.9 [41.6, 42.3]

Note: CI = confidence interval

Table 2 depicts the top ten presenting complaints and the corresponding vital signs information. The top ten complaints are fever (12.2% of total sample), non-head/face/neck injuries (11.4%), abdominal pain (8.9%), chest pain (7.8%), head/neck/face injuries (5.0%), vomiting (4.1%), headache (3.7%), shortness of breath (3.3%), back pain (3.2%) and diarrhea (2.3%). The most commonly available vital signs for these specific presenting complaints were blood pressure (27.8% of all ten top ten presenting complaints), pulse (25.8%) and temperature (24.0%). Important vital signs pertinent to each symptom were absent to varying extents; for example, temperature information was not available for 61.3% of patients with fever as a presenting complaint, and respiratory rate and oxygen saturation were not recorded in 83.3% patients presenting with a chief complaint of shortness of breath.

**Table 2:** Frequency of vital signs for top ten presenting complaints (patients >12 years)

Presenting complaints	N (% of total sample n = 259288)	*Pulse	*BP	* T°C	*GCS	*RR	*SpO_2_
		
		N (% of each presenting complaints)					
Fever	31554 (12.2)	9964 (31.6)	9645 (30.6)	12528 (39.7)	1170 (3.7)	3797 (12.0)	1288 (4.1)
Injury (non-head/face/neck)	29695 (11.4)	2767 (9.3)	3200 (10.8)	2583 (8.7)	441 (1.5)	2267 (7.6)	1437 (4.8)
Abdominal pain	23170 (8.9)	5886 (25.4)	5986 (25.8)	4516 (19.5))	311 (1.3)	1520 (6.6)	732 (3.2)
Chest pain	20130 (7.8)	8630 (42.9)	9076 (45.1)	7008 (34.8)	1371 (6.8)	5157 (25.6)	1409 (7.0)
Injury (Head/face/neck)	13309 (5.0)	1769 (13.3)	2064 (15.5)	1669 (12.5)	394 (3.0)	1418 (10.7)	852 (6.4)
Vomiting	10629 (4.1)	3135 (29.5)	3372 (31.7)	2508 (23.6)	211 (2.0)	1098 (10.3)	606 (5.7)
Headache	9516 (3.7)	3267 (34.3)	3706 (38.9)	2779 (29.2)	286 (3.0)	887 (9.3)	271 (2.8)
Shortness of breath	8548 (3.3)	2711 (31.7)	3087 (36.1)	1936 (22.6)	219 (2.6)	1436 (16.8)	611 (7.2)
Back pain	8239 (3.2)	1557 (18.9)	1597 (19.4)	1392 (16.9)	39 (0.5)	368 (4.5)	169 (2.1)
Diarrhea	5954 (2.3)	1835 (30.8)	1916 (32.2)	1588 (26.7)	108 (1.8)	608 (10.2)	244 (4.1)
Total	160744 (62.0)	41521 (25.8)	43649 (27.8)	38507 (24.0)	4550 (2.8)	18556 (11.5)	7619 (4.7)

Note:

• BP = SBP (Systolic Blood Pressure) & DBP (Diastolic Blood Pressure); T°C = temperature in Celsius; GCS = Gaslow Coma Scale; RR = Respiratory Rate; SpO_2 _= Oxygen saturation

• * Number of cases (percentage) with available information

• Bolded cells indicate critical information pertinent to case presentation

Table 3 presents the distribution of high-acuity patients based on the presence of a priori defined abnormal vital signs and selected presenting complaints. When abnormal clinical signs were matched with the presenting conditions, we were able to identify 12.8% patients with an abnormal shock index (SI), and abnormal temperature (26.4%) who presented with fever. Similarly, 13.3% patients with chest pain, 12.9% with diarrhea and 10.2% patients with vomiting were found to be hemodynamically unstable, as indicated by an abnormal SI. The Glasgow coma score (GCS) was very infrequently recorded even among patients with closed head injuries and headaches (3.0% and 3.0% respectively). (Table 2) Only 11.6% patients with shortness of breath had any information related to respiratory rate or oxygen saturation.

**Table 3:** High-Acuity Patients in the Pak-NEDS Study, Based on Vital Signs and Selected Presenting Complaints (patients >12 years)

Presenting complaints	N (% of total sample n = 259288)	Abnormal Shock Index	Low GCS or Altered Consciousness	Abnormal Temperature	Abnormal RR or abnormal SpO_2_	Very High Blood Pressure
		
		(N, % of each complaint)				
Fever	31554 (12.2)	4050 (12.8)	--	8319 (26.4)	--	--
Abdominal pain	23170 (8.9)	1945 (8.4)	--	--	--	--
Chest pain	20130 (7.8)	2678 (13.3)	--	--	--	69 (0.3)
Vomiting	10629 (4.1)	1084 (10.2)	--	--	--	--
Diarrhea	5954 (2.3)	765 (12.9)	--	--	--	--
Back pain	8239 (3.2)	--	--	--	--	22 (0.3)
Head/face/neck injury	13309 (5.0)	--	746 (5.6)	--	--	--
Headache	9516 (3.7)	--	188 (2.0)	--	--	--
Shortness of breath	8548 (3.3)	--	--	--	994 (11.6)	--

Note:

• GCS = Gaslow Coma Scale; RR = Respiratory Rate; SpO_2 _= Oxygen saturation

• Ranges for reported vital signs in this table are as follows: Abnormal shock index="shock index<0.5 or >0.7"; Low GCS="3≤GCS≤12"; Abnormal temperature="<36.5°C (97.8 °F) or > 38°C (100.4 °F)"; Abnormal RR="RR<12/min or >18/min"; Abnormal SpO_2 _= "SpO_2 _≤94%"; Very high blood pressure="Systolic Blood Pressure >180 mm/Hg"

**Figure 2:**
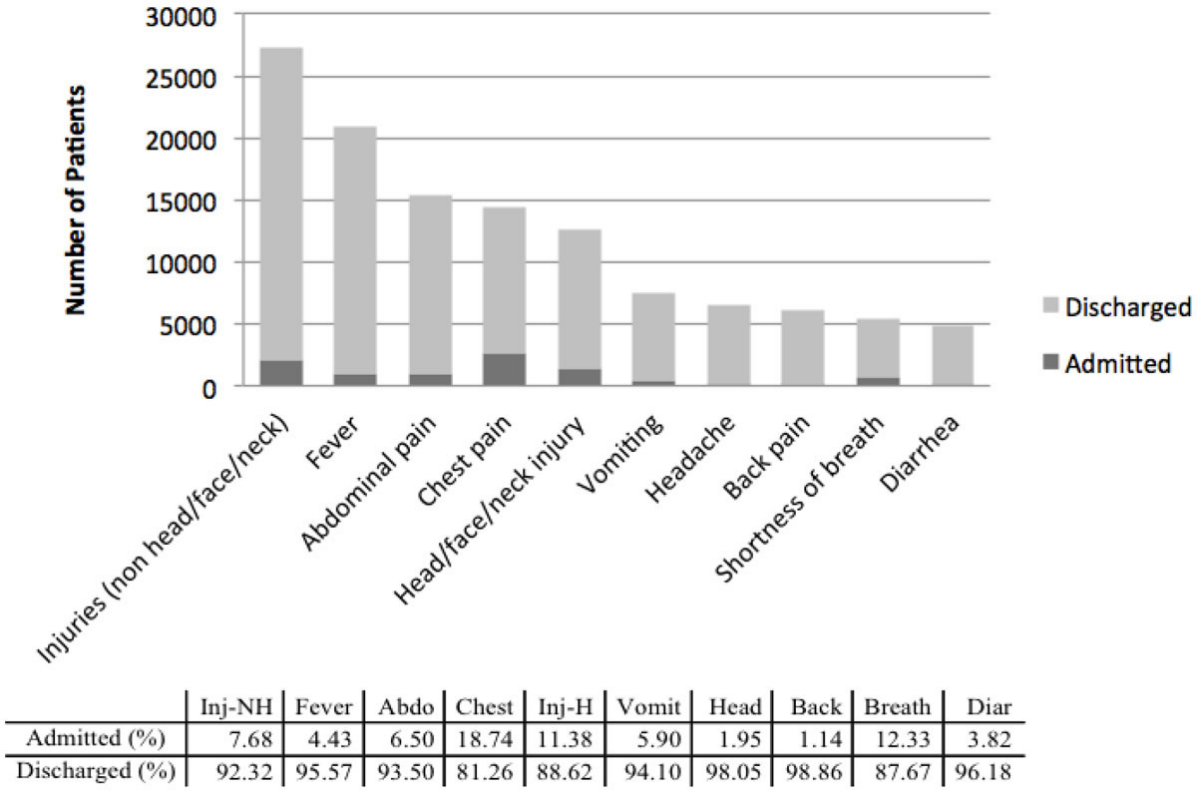
**Admission disposition of the top ten presenting complaints in Pakistan National Emergency Department Surveillance study (n = 259,288)**. Note: Inj-NH = injuries (non head/face/neck); Abdo = abdominal pain; Chest = chest pain; Inj-H = head/face/neck injury; Vomit = vomiting; Head = headache; Back = back pain; Breath = shortness of breath; Diar = diarrhea.

Figure 2 depicts the distribution and final disposition of the top 10 complaints in patients with either normal or abnormal vital signs. With respect to disposition from the ED, the majority of patients with fever, vomiting and diarrhea were discharged (95.6%, 94.1%, 96.2%, respectively). A larger proportion of patients with chest pain (18.7%), shortness of breath (12.3%), and head injury (11.4%) were admitted for further workup and treatment.

In Table 4 the hospital admission percentage was separately presented for patients with normal vital signs and those with abnormal vital signs. The admission percentage is higher for patients with any abnormal signs who presented with fever, abdominal pain, chest pain, vomiting, headache, shortness of breath, back pain and diarrhea (P < 0.01 respectively).

**Table 4:** Disposition of top ten presenting compliant patients with normal or abnormal vital signs (patients >12 years)

Presenting complaints	N	Vital sign available	Normal vital sign	Admitted	Abnormal vital sign	Admitted	P-Value***
		N (%)*	N (%)*	N (%)**	N (%)*	N (%)**	

Fever	31554	16800 (53.2)	6347 (20.1)	**139 (2.2)**	10453 (33.1)	**443 (4.2)**	0.000
Injury (non-head/face/neck)	29695	5786 (19.5)	3280 (11.0)	**297 (9.1)**	2506 (8.4)	**193 (7.7)**	0.067
Abdominal pain	23170	15947 (68.8)	12014 (51.9)	**252 (2.1)**	3933 (17.0)	**195 (5.0)**	0.000
Chest pain	20130	9921 (49.3)	4931 (24.5)	**545 (11.1)**	4990 (24.8)	**998 (20.0)**	0.000
Injury (Head/face/neck)	13309	3175 (23.9)	1523 (11.4)	**156 (10.2)**	1652 (12.4)	**187 (11.3)**	0.329
Vomiting	10629	6586 (62.0)	4415 (41.5)	**87 (2.0)**	2171 (20.4)	**95 (4.4)**	0.000
Headache	9516	5380 (56.5)	3201 (33.6)	**39 (1.2)**	2179 (22.9)	**53 (2.4)**	0.001
Shortness of breath	8548	4915 (57.5)	3462 (40.5)	**153 (4.4)**	1453 (17.0)	**250 (17.2)**	0.000
Back pain	8239	6342 (77.0)	5206 (63.2)	**17 (0.3)**	1136 (13.8)	**15 (1.3)**	0.000
Diarrhea	5954	3725 (62.6)	2645 (44.4)	**44 (1.7)**	1080 (18.1)	**54 (5.0)**	0.000

Note: Abnormal vital sign include the presence of any of the following conditions: Abnormal shock index="shock index<0.5 or >0.7"; Low GCS="3≤GCS≤12"; Abnormal temperature="<36.5°C (97.8 °F) or > 38°C (100.4 °F)"; Abnormal RR="RR<12/min or >18/min"; Abnormal SpO_2 _= "SpO_2 _≤94%"; Very high blood pressure="Systolic Blood Pressure >180 mm/Hg"

* %: The percentage of available vital sign, normal vital sign, and abnormal vital sign compare to total sample size of each presenting complaint

** %: The percentage of admitted patients within the number of patients with normal or abnormal vital sign

***: P-value of chi-squared test to compare admission between patients with normal vital signs and those with abnormal vital signs

## Discussion

In our study we found that that most of the critical information required for simple assessment among common clinical presentations in the EDs of Pakistan was missing. Only 11.6% patients with shortness of breath had any information related to abnormal respiratory rate or oxygen saturation, which suggests that the current decision-making was likely based on criteria that did not include considerations for vitals. The recording of ED vital signs is critical to identifying and treating those who need care first, thus maximizing the use of available resources for patient benefit and minimizing time to definitive treatment [6,17,18]. Most urban tertiary centers in developing countries are overwhelmed with high volumes of semi-urgent conditions, and are facing resource constraints for critically ill patients [10,11,19]. Therefore, vital signs checked at the initial assessment along with other basic clinical information - often termed as "triage" -- is helpful in directing ED resources appropriately. Essential information such as signs of physiological deterioration work as a supplement to presenting symptoms, which allows for easier decision-making [20].

In places with more healthcare resources, there are often several levels of care available, such as intensive care units, step-downs, monitored beds, regular floors etc., and admission decisions to these units often takes into consideration the abnormal vital signs. In many hospitals in Pakistan, ICU beds are severely restricted and the disposition in the hospital is usually to one single type of care area (termed "general ward" in Pakistan), thus making vital signs less critical for admission decisions. On the contrary, lack of objective data may lead to poor assessment and delays in intervention. The result might be over- or underestimation the severity of illness, with potential downstream effects on patient outcomes, ED crowding and utilization of health services [21-23]. In the current study we found that only one private hospital had a formal triage system in place and the rest of them operate on first come first served basis.

Another important finding in the study was the correlation of admission decisions with abnormal vital signs for the majority of common complaints except injuries. Subtle signs such as abnormal heart and respiratory rates can predict critical care admissions in many patients with common complaints such as nausea, vomiting and diarrhea [26]. Other types of information are also useful in making early clinical decisions. For patients with chest pain, an electrocardiogram (EKG) can identify those with serious medical conditions. The shock index, a numeric derivative from the blood pressure and pulse rate, can also help to prioritize patients and reduce adverse clinical outcomes in a select group, such as those with trauma or suspected hypovolemia [24]. Young patients suffering from hypovolemia do not demonstrate hypotension and signs of clinical shock until late, and calculating the SI might be helpful in differentiating such patients [24,25].

The findings of this study point towards a large gap in the quality of care in the large urban EDs of Pakistan and highlight the need for the development of national guidelines for the assessment and categorization of ED patients. The introduction of an essential list of data points required for ED patients, providing the staff with necessary equipment and supplies, as well as training, can help the frontline healthcare personnel [or staff] perform tasks such as manually taking blood pressure, assessing level of consciousness, recording pulse and oxygen saturation through a pulse oximeter.

Studies done in high income countries demonstrate that vital signs may lengthen the triage procedure and many times vital signs are omitted to keep up with the gatekeeping of the EDs [27]. This could be more challenging in an environment where the general public attending the hospitals is not familiar with the triage process and its impact on decision-making. Nevertheless, these simple measures may actually improve the patients' confidence towards the ED personnel and help create a database which can be utilized for performance assessment, outcome studies, and utilization of resources in similar resource-poor settings [28-30].

This study also has a number of limitations. Our data did not capture sufficient seasonal variations, patients' care-seeking behaviors and other important factors such lack of primary care facilities and social support system, which may have a bearing on clinical decision-making and ED disposition. Also, high turnover of ED patients in those hospitals may have some bearing on vital signs documentation, which is a significant limitation in generalizability of the results. Similarly, the shock index can be used to identify a set of patients with clinically serious illnesses, but some patients with other symptoms such as shortness of breath, impaired consciousness and chest pain could have normal indices. In addition, although the Pak-NEDS carefully selected seven hospitals in five provinces of Pakistan, these represented large teaching hospitals and may not represent other types of hospitals or those located in rural areas.

## Conclusion

In the majority of patients presenting to the large urban EDs, vital signs were not documented anytime during patients visit to the ED. The presence of abnormal vital signs correlated with admission disposition for the majority of common complaints. This study calls for a need to develop and implement national guidelines for assessment and prioritization of patients presenting to emergency departments in Pakistan.

## Competing interests

The authors declare that they have no competing interests.

## Authors' contributions

AM conceptualized the study, and wrote the first draft of manuscript and all the revisions. SH conducted the data analysis and helped in manuscript writing. WZ and NB helped in critical review of the first draft. FAS was local collaborator of Pak-NEDS in Quetta and provided critical review of the draft. JAR conceptualized Pak-NEDS, was involved in the implementation, data collection and analysis procedures and provided critical review and final approval of the manuscript.
